# Post-breast Reduction Pyoderma Gangrenosum Managed With Gender-Affirming Top Surgery

**DOI:** 10.7759/cureus.20777

**Published:** 2021-12-28

**Authors:** Abigail E Peoples, Ivo A Pestana

**Affiliations:** 1 Plastic and Reconstructive Surgery, Wake Forest Baptist Medical Center, Winston-Salem, USA

**Keywords:** postoperative pyoderma gangrenosum, nipple-sparing mastectomy, plastic surgery, gender affirmation surgery, breast reduction

## Abstract

Pyoderma gangrenosum (PG) is a rare skin condition characterized by ulcerative lesions and most often associated with inflammatory bowel diseases (IBDs), ulcerative colitis, or Chron’s disease. Postsurgical pyoderma gangrenosum (PSPG) of the breast is exceptionally rare and can occur in the absence of IBD. We report on a patient with pyoderma gangrenosum following elective breast reduction and subsequent reconstruction with gender-affirming top surgery. Through discussion of this IRB-reviewed case, we encourage reconstructive surgeons to incorporate understanding of a patient’s gender identity, expression, or goals during treatment to optimize their patient-centered care.

## Introduction

Pyoderma gangrenosum (PG) is a noninfectious, inflammatory skin disease (neutrophilic dermatosis) characterized by the development of painful ulcers that progressively worsen. Although first described almost a century ago, the standard of care for this condition remains unknown, and a majority of management plans are empiric. Postsurgical pyoderma gangrenosum (PSPG) of the breast is exceptionally rare, with an estimated incidence of 3-10 cases per million people in the United States and Europe [1-5]. Consequently, understanding of the disease process has primarily been through case reports and case series. We report on a patient with pyoderma gangrenosum following elective breast reduction and subsequent reconstruction with gender-affirming top surgery.

## Case presentation

**Figure 1:**
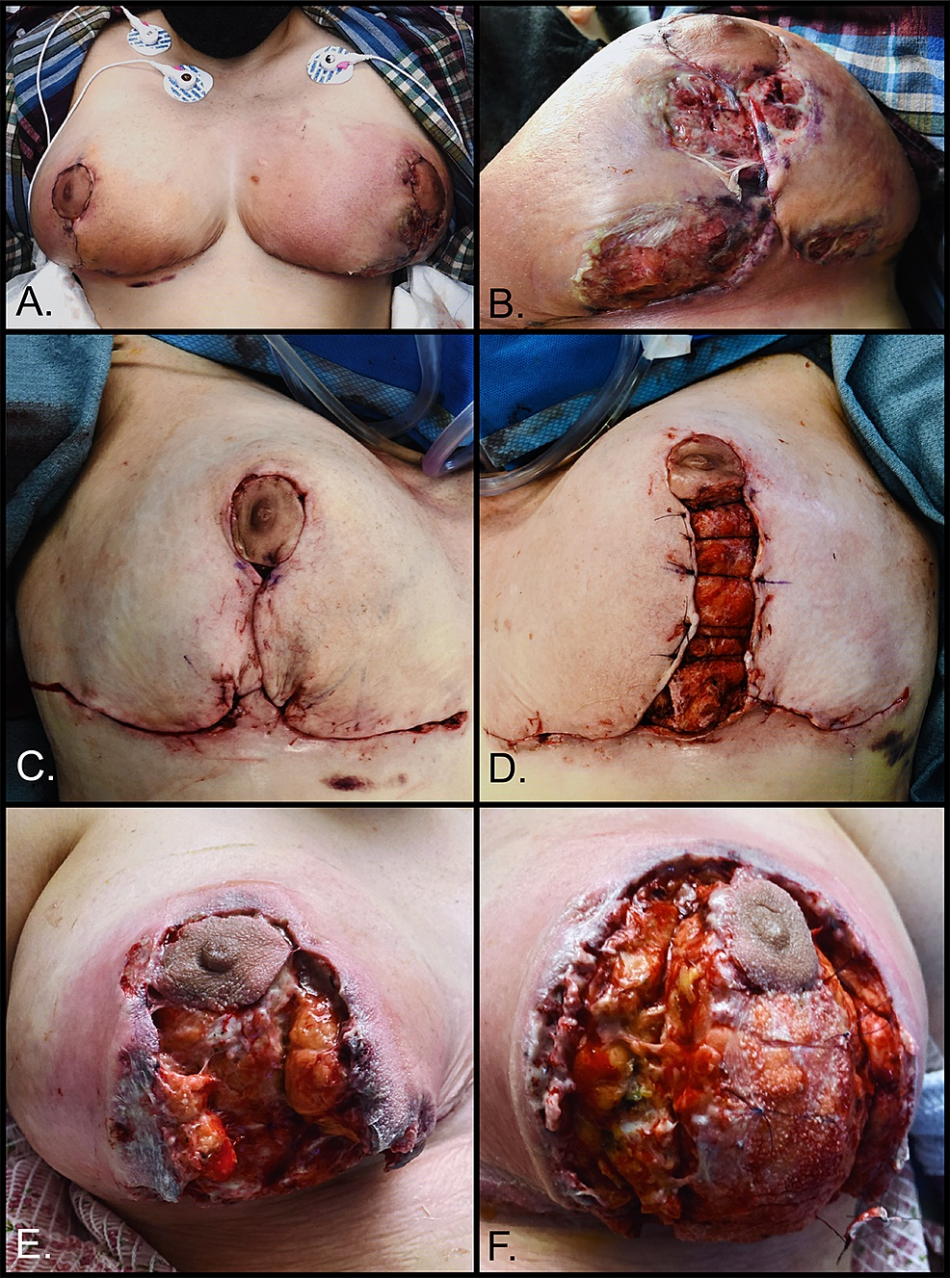
A: Bilateral breasts at initial presentation. B: Left breast at initial presentation. C: Right breast after final debridement operation. D: Left breast after final debridement operation. E: Right breast on postoperative day four following final debridement. F: Left breast on postoperative day four following final debridement.

The patient is a 25-year-old female with polycystic ovarian syndrome (PCOS), fibromyalgia, and postural orthostatic tachycardia syndrome (POTS) who developed bilateral breast wounds three days postoperatively from elective breast reduction surgery. She was initially managed by the operative surgeon with oral antibiotics and wound care without improvement and then transferred to our emergency department (ED) on postoperative day six due to concern for a necrotizing infection. On evaluation in the ED, the patient was noted to have a low-grade fever, tachycardia, and leukocytosis associated with bilateral purple-rimmed breast wounds inferior to the preserved nipple-areolar complexes (Figure 1). Erythema of the breasts, left greater than right, was also noted with mild expressible purulent material from the left areolar complex inset. For the working diagnosis of bilateral breast necrotizing infection, the patient was taken emergently to the operating suite for debridement. Operative findings demonstrated mild amounts of necrotic tissue and minimal purulence other than that expressed in the emergency department. Two subsequent operative debridements were completed over a 72-hour period, and all gram stains and blood and wound cultures demonstrated no bacterial or fungal growth. At the third operative debridement, tissue biopsies were taken, all demonstrative of pyoderma gangrenosum. The diagnosis of PG was finalized in consultation with infectious disease and dermatology. With the initiation of steroid therapy, wounds showed improvement. The patient was discharged with plans for continued outpatient wound care.

**Figure 2:**
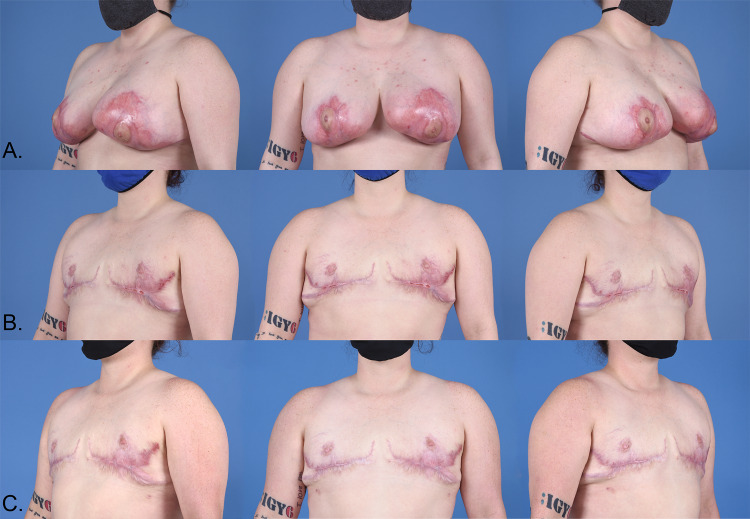
Row A: Healing bilateral PSPG wounds prior to mastectomy. Row B: Status post-mastectomy with free nipple graft. Row C: Post revision with fat grafting and tissue rearrangement.

Following several months of wound care therapy and hyperbaric oxygen therapy (HBOT), the patient sought reconstructive surgery due to pain associated with her healing breast wounds and resultant breast asymmetry. Due to her gender identity, gender-affirming top surgery was discussed as a reconstructive option. During this consultation, the patient confirmed that her goal, the alteration of her chest to a more masculine appearance, was inadequately achieved with breast reduction. Approximately four months after initial mammoplasty reduction, gender-affirming female-to-male (FTM) top surgery was completed with nipple-sparing mastectomy using the free nipple graft technique. Perioperatively, the patient was treated with immunomodulatory medications: 10 mg prednisone (Deltasone, Pfizer) and 100 mg cyclosporine (Gengraf, AbbVie Inc.) daily. Postoperative convalescence was normal, and two months later, a revision with suction-assisted lipectomy of bilateral axillary lipodystrophy was completed to improve chest contour (Figure 2). To date, the patient has had no signs of recurrent PG and is satisfied with her surgical outcome.

## Discussion

Postsurgical pyoderma gangrenosum is a rare complication of breast surgery associated with significant morbidity due to delayed diagnosis. PSPG symptoms often manifest within the week following surgical intervention. The mean time from PSPG symptom onset to correct diagnosis is 12.5 days, although cases have gone undiagnosed for as long as three years [1].

Due to the rarity of PSPG, systematic and literature reviews have been instrumental in the diagnosis and treatment of PG. The most recent reviews of PG cases emphasized the importance of early diagnosis and avoidance of wound debridement to minimize the risk of permanent deformity. Accurate and efficient diagnosis is challenging as PG mimics severe cellulitis or necrotizing infection. Understandably, patients with PG are often initially managed with irrigation and debridement, which may exacerbate PSPG. A systematic review by Ehrl et al. analyzed 87 cases of pyoderma gangrenosum of the breast and proposed major and minor criteria for diagnosing PG. The major criteria needed were the presence of painful, progressive, violaceous, irregular ulcers [1]. In addition, suspicion for PG should be elevated in the context of a presumed necrotizing infection that yields no positive culture results, lesions that spare the nipple-areolar complex, and ulcerated areas that do not cross suture lines [1].

PG is a diagnosis of exclusion that often follows repeated, failed debridements. For patients with delayed diagnosis, repeated wound debridement can lead to soft tissue defects that require complex reconstruction. If reconstructive procedures are needed following acute PG, consideration of perioperative immunomodulatory administration to avoid further pathergy is essential [1]. Even with early diagnosis and treatment, patients with PG may have a protracted wound healing course. No single regimen has been identified as superior in the treatment of PG, but most successful treatment regimens and perioperative prophylaxis include corticosteroids, cyclosporine, or a combination of both [6]. In the absence of PG improvement with these medications, other immunomodulatory agents (e.g., monoclonal antibodies) can be considered [7]. Treatment is continued until the resolution of ulcers, which can take weeks to months.

Central to the optimal reconstructive outcome, in this case, was a treatment plan acknowledging the multifaceted, individualized nature of gender identity and expression. Research in the fields of psychology, neuroscience, and neuroendocrinology demonstrate an evolving understanding of gender identity that challenges the gender binary [8]. While a growing body of research supports the importance of gender-affirming care for transgender patients [9], the language surrounding this care continues to indicate a gender binary. Top surgery is categorized as female-to-male (FTM) or male-to-female (MTF), suggesting patients simply desire to transition from one sex to another. Further understanding of gender indicates that gender identity and expression are exceptionally individualized [10]. Consequently, top surgery is a procedure that should not be considered solely in patients who are pursuing transition but may be considered in patients who feels their current chest structure is incongruent with their ideal gender expression or would incur medical benefit from the procedure. Exploration of the ideal outcome for our patient yielded their desire for breast removal. Gender-affirming top surgery was likely not offered or understood as an option for our patient when initially discussing reconstructive procedures as they did not explicitly seek transition care. Yet, early in their complicated postoperative course, our patient inquired about mastectomy. This inquiry occurred almost two months before top surgery was offered as a treatment option by a patient-driven consultation with another reconstructive breast surgeon. While our patient was not seeking transition-related care, they did desire mastectomy and top surgery for a medical and aesthetic outcome congruent with their goals and compatible with their individual gender expression.

## Conclusions

While rare, PSPG should always be included in the differential diagnosis of postoperative wounds, especially when necrotizing infection is on the differential. Although histological findings are not pathognomonic for PG, a biopsy of wounds requiring multiple debridement procedures would be prudent. When PG is diagnosed, early initiation of immunomodulatory medications and judicious debridement are imperative. As our understanding of gender continues to expand, we encourage reconstructive surgeons to incorporate an understanding of a patient’s gender identity, expression, or goals during treatment to optimize their patient-centered care.

## References

[1] Ehrl DC, Heidekrueger PI, Broer PN (2018). Pyoderma gangrenosum after breast surgery: a systematic review. J Plast Reconstr Aesthet Surg.

[2] Zelones JT, Nigriny JF (2017). Pyoderma gangrenosum after deep inferior epigastric perforator breast reconstruction: systematic review and case report. Plast Reconstr Surg Glob Open.

[3] Tuffaha SH, Sarhane KA, Mundinger GS (2016). Pyoderma gangrenosum after breast surgery: diagnostic pearls and treatment recommendations based on a systematic literature review. Ann Plast Surg.

[4] Larcher L, Schwaiger K, Eisendle K, Ensat F, Heinrich K, di Summa P, Wechselberger G (2015). Aesthetic breast augmentation mastopexy followed by post-surgical pyoderma gangrenosum (PSPG): clinic, treatment, and review of the literature. Aesthetic Plast Surg.

[5] Zuo KJ, Fung E, Tredget EE, Lin AN (2015). A systematic review of post-surgical pyoderma gangrenosum: identification of risk factors and proposed management strategy. J Plast Reconstr Aesthet Surg.

[6] Haag CK, Bacik L, Latour E, Morse DC, Fett NM, Ortega-Loayza AG (2020). Perioperative management of pyoderma gangrenosum. J Am Acad Dermatol.

[7] Ahronowitz I, Harp J, Shinkai K (2012). Etiology and management of pyoderma gangrenosum: a comprehensive review. Am J Clin Dermatol.

[8] Hyde JS, Bigler RS, Joel D, Tate CC, van Anders SM (2019). The future of sex and gender in psychology: five challenges to the gender binary. Am Psychol.

[9] American Medical Association (2021). American Medical Association: Health insurance coverage for gender-affirming care of transgender patients. https://www.ama-assn.org/system/files/2019-03/transgender-coverage-issue-brief.pdf.

[10] Diamond LM (2020). Gender fluidity and nonbinary gender identities among children and adolescents. Child Dev Perspect.

